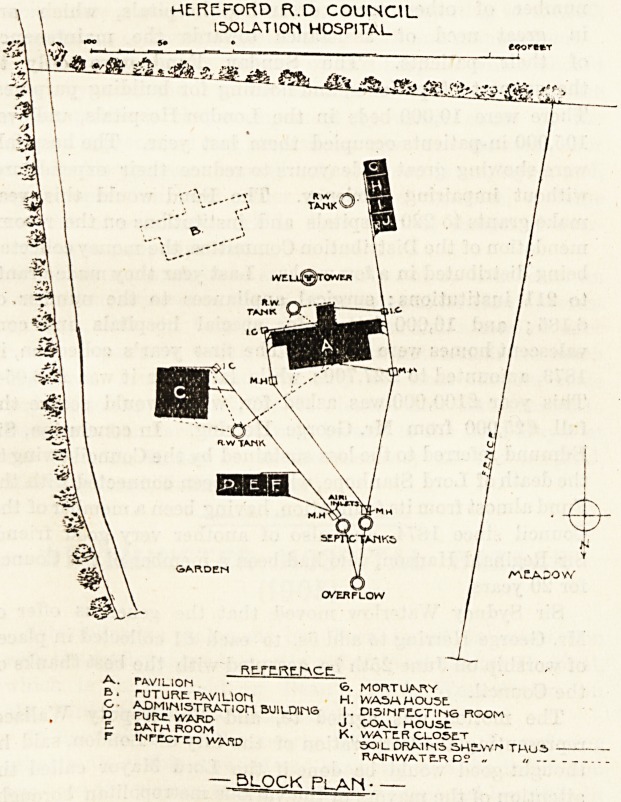# Hereford Isolation Hospital

**Published:** 1905-05-13

**Authors:** 


### HEREFORD ISOLATION HOSPITAL.

This hospital, which has recently been erected by the Hereford Rural District Council, stands on a plot of land three acres and a half in extent, and about four miles from the City of Hereford. The site is an elevated one overlooking a well-wooded district, and it is sufficiently removed from any high road to minimise the risk of infection spreading in the neighbourhood. A well 80 feet deep was sunk on the estate, and an abundant supply of pure water was found. The water is raised by a windmill having a steel tower 100 feet high, on which is a storage or supply-tank for the distribution of water to the various divisions of the hospital.

At present the building consists of four blocks, and there is ample room for the erection of a fifth without any component part of the hospital being within 40 feet of its fellow, or within 50 feet of the boundary fence.

The isolation pavilion faces nearly south. It consists of a central part and two wings. The entrance is to the north, and on the right on entering is the bath-room, and on the left is the store-room. A passage runs at the south of these, and on the right this gives access to the male ward, and on the left to the female ward. These wards are exactly alike. Each is 25 feet square, 13 feet high, and each contains four beds. Each bed has a window on both sides, and the floor space is 150 square feet per bed, and the cubic space is upwards of 2,000 feet per bed. The wall space is 12 feet 6 inches per bed. The sanitary annexes are placed at the south-east and south-west angles respectively, and they are properly constructed with cross-ventilated passages. The nurses' duty-room is to the south, and is placed between the wards so that it commands both of them, and there are inspection windows for the better observation of the patients.

This room is partly fitted up as a kitchen, and the block containing the scullery and pantry projects to the south for about 14 feet. This is an arrangement which we should be disposed to criticise adversely, because, during the winter months when the sun is low and sunshine most needed, the projection will keep the morning sun out of the men's ward and the afternoon sun out of the women's ward.

The inside of the walls are covered with silicon plaster; all the corners have been rounded off, and all mouldings, architraves, and projections of all kinds have been studiously avoided. The warming is carried out by the best of all means—open fireplaces. The window space is ample for both light and ventilation, and some of the windows are made with a section opening inwards. The floors of this pavilion block are of cement concrete.

East of the pavilion is the administrative block, containing sitting-rooms, kitchen, scullery, pantry, stores, and four bedrooms. Similar precautions against the accumulation of dust have been taken in this block as in the pavilion, and therefore the result cannot fail to be an eminently sanitary abode.

Towards the south-west corner is the range of buildings containing the mortuary, washhouse, disinfecting room, etc., and towards the north-east corner is the block containing the "infection room," the "pure room," and the bathroom. It is stated that this, which is really the discharging block, has the advantage of four buildings concentrated into one, and has been provided to serve many purposes—namely, an observation ward, an isolation ward for any virulent case, a "pure" ward for retaining a patient prior to returning home, and an ordinary discharging block. We hope the architect is not expecting too much.

All the blocks are built of red bricks, relieved with string courses of buff bricks. The roofs are covered with Broseley tiles. Special attention seems to have been paid to the drainage. Extraction shafts are carried up to the highest point of each block; there are manholes at every change of direction in the drains; the sewage flows into air-tight septic tanks; and the drains from the pavilion block have been kept separate from the others. Another commendable feature is the provision for the storage of rain water. East block has its own underground tank, and its own pumping arrangements attached thereto.

The cost of the buildings was £2,050, which appears to us a moderate sum considering the amount and quality of the work. The architect was Mr. E. G. Davies, of Hereford, and the contractor was Mr. C. Cooke.

## Figures and Tables

**Figure f1:**
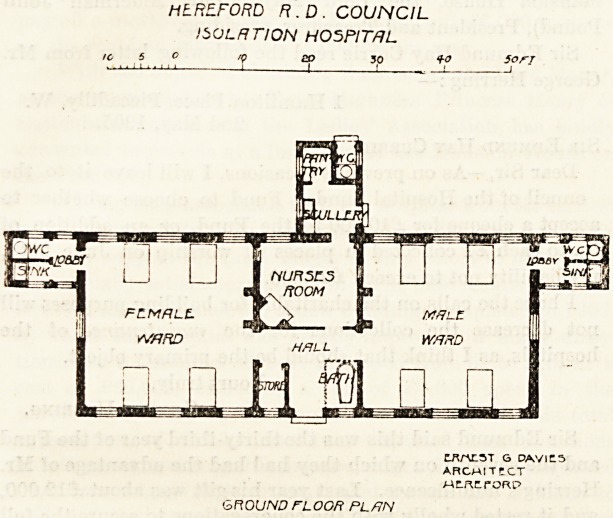


**Figure f2:**